# Proteomics of Host-Helminth Interactions

**DOI:** 10.3390/pathogens10101317

**Published:** 2021-10-13

**Authors:** Mark W. Robinson, Krystyna Cwiklinski

**Affiliations:** 1 School of Biological Sciences, Queen’s University Belfast, 19 Chlorine Gardens, Belfast BT9 5DL, Northern Ireland, UK; 2 Center of One Health (COH) and Ryan Institute, School of Natural Science, National University of Ireland Galway, H91 DK59 Galway, Ireland

Helminth infections in people contribute to the 1.31 billion cases of neglected tropical diseases and malaria worldwide, representing a loss of 10.6 million disability adjusted life years each year [1]. The diseases caused by these parasites are endemic in over 100 countries where they typically affect people living in poverty and, accordingly, the World Health Organisation recognises them as some of the world’s most neglected tropical diseases [2,3]. Helminth parasites also have a major negative impact on animal production and welfare, accounting for >55% of all farm animal disease and approximately 17% of animal production losses [4,5].

With their complex life cycles and varied developmental stages, each with distinct biology and host interactions, effective treatment of helminth infection can be challenging. For most species, we are reliant on only a few drugs (e.g., praziquantel and ivermectin), but these do not prevent reinfection and require repeated usage. Indeed, this is not sustainable in the long term, due to the threat of drug resistance, and has led to considerable research effort toward the development of first-generation helminth vaccines (reviewed by Claerebout and Geldhof, 2020 [6] and Perera and Ndao, 2021 [7]). This has not been straightforward and has indeed been hampered by the striking ability of helminths to modulate host immune responses [8,9,10]. It has become evident that new strategies to prevent and treat helminth infections are dependent on a deeper understanding of helminth–host interactions. A major task is to identify virulence factors/immunomodulatory molecules that are essential for parasite survival which may serve as novel vaccine candidates or as targets for anthelmintic drugs. Accordingly, mass spectrometry-based proteomics techniques, which have revolutionised our ability to identify helminth proteins over the past 20 years, will continue to have a key role in this process.

In this Special Issue, which focuses on the proteomics of host-helminth interactions, there are nine timely contributions which highlight recent advances in this field but also bring into focus some research priorities moving forward. The collection includes four research papers. The first of these, by Długosz et al., focuses on the zoonotic nematode *Toxocara* canis [11]. In this study the authors used a yeast two-hybrid assay to identify human proteins that may directly interact with *T. canis* antigens. As such, the study provides an initial basis for understanding the immunological and pathological effects of this infection in humans. Next, Wititkornkul et al. examine the immunomodulatory potential of extracellular vesicles (EVs) released by the equine tapeworm *Anoplocephala perfoliata* [12]. EVs have emerged as novel vehicles for transfer of helminth-derived molecules to host cells, and, by describing the cargo of *A. perfoliata* EVs, this paper uncovers potential mechanisms of parasite-to-host communication during infection. The next research paper by Lan et al., together with that of Becerro-Recio et al., focuses on the identification of antigens secreted by the liver fluke, *Fasciola hepatica*, that are recognised by the host immune response [13,14]. Here, readers will be able to compare and contrast the different experimental approaches taken by these authors (co-immunoprecipitation vs. two-dimensional Western blot, both coupled with mass spectrometry) to profile antigenic molecules shed by the liver fluke during infection of sheep.

The next five articles are reviews covering the interactions of nematode and trematode parasites with their mammalian hosts. Thiangtrongjit et al. discuss how profiling the secretome of *Gnathostoma spinigerum*, a zoonotic nematode infection, coupled with immunoproteomics approaches, is driving the development of novel diagnostic tools for human gnathostomiasis [15]. The next review article by Carson and Gobert highlights how recent comparative proteomics analysis has advanced our understanding of the antigens released by schistosome eggs and how these drive immunological and pathological responses in host tissue [16]. The next two review articles by Wang and Gasser and Bennett and Robinson summarise progress in the use of mass spectrometry-based proteomics techniques to study nematode and trematode parasites, respectively. With an emphasis on emerging technologies, both reviews pin-point key research areas that will enhance our understanding of host–helminth interactions with a view toward new methods of treatment and diagnosis [17,18]. Finally, Porras-Silesky et al. review the current understanding of how the nematode parasite, *Spirocerca lupi*, induces cancer in dogs [19]. By discussing the role of specific secreted antigens from other carcinogenic helminths (such as *Clonorchis sinensis, Opisthorchis viverrini* and *Schistosoma haematobium*, and other lesser studied species) they propose a mechanism for *S. lupi*-induced cancer in dogs which is underpinned by sustained inflammatory responses to parasite antigens.

In summary, by inviting articles from leading helminth researchers, the aim of this Special Issue was to highlight how mass spectrometry-based proteomics has advanced our understanding of the host–parasite interaction. We hope that the articles compiled will stimulate discussion and further research in this area and contribute to the development of new strategies for helminth diagnosis and control.
